# HIV Clustering in Mississippi: Spatial Epidemiological Study to Inform Implementation Science in the Deep South

**DOI:** 10.2196/publichealth.8773

**Published:** 2018-04-03

**Authors:** Thomas J Stopka, Lauren Brinkley-Rubinstein, Kendra Johnson, Philip A Chan, Marga Hutcheson, Richard Crosby, Deirdre Burke, Leandro Mena, Amy Nunn

**Affiliations:** ^1^ Department of Public Health and Community Medicine School of Medicine Tufts University Boston, MA United States; ^2^ Clinical and Translational Science Institute Tufts University School of Medicine Boston, MA United States; ^3^ Department of Social Medicine University of North Carolina Chapel Hill, NC United States; ^4^ Center for Health Equity Research University of North Carolina Chapel Hill, NC United States; ^5^ Mississippi State Department of Health Jackson, MS United States; ^6^ School of Public Health Brown University Providence, RI United States; ^7^ Department of Medicine Brown University Providence, RI United States; ^8^ College of Public Health University of Kentucky Lexington, KY United States; ^9^ John D Bower School of Population Health University of Mississippi Medical Center Jackson, MS United States; ^10^ Rhode Island Public Health Institute Providence, RI United States

**Keywords:** hotspots, HIV, racial disparities, social determinants of health, HIV treatment, HIV screening

## Abstract

**Background:**

In recent years, more than half of new HIV infections in the United States occur among African Americans in the Southeastern United States. Spatial epidemiological analyses can inform public health responses in the Deep South by identifying HIV hotspots and community-level factors associated with clustering.

**Objective:**

The goal of this study was to identify and characterize HIV clusters in Mississippi through analysis of state-level HIV surveillance data.

**Methods:**

We used a combination of spatial epidemiology and statistical modeling to identify and characterize HIV hotspots in Mississippi census tracts (n=658) from 2008 to 2014. We conducted spatial analyses of all HIV infections, infections among men who have sex with men (MSM), and infections among African Americans. Multivariable logistic regression analyses identified community-level sociodemographic factors associated with HIV hotspots considering all cases.

**Results:**

There were HIV hotspots for the entire population, MSM, and African American MSM identified in the Mississippi Delta region, Southern Mississippi, and in greater Jackson, including surrounding rural counties (*P*<.05). In multivariable models for all HIV cases, HIV hotspots were significantly more likely to include urban census tracts (adjusted odds ratio [AOR] 2.01, 95% CI 1.20-3.37) and census tracts that had a higher proportion of African Americans (AOR 3.85, 95% CI 2.23-6.65). The HIV hotspots were less likely to include census tracts with residents who had less than a high school education (AOR 0.95, 95% CI 0.92-0.98), census tracts with residents belonging to two or more racial/ethnic groups (AOR 0.46, 95% CI 0.30-0.70), and census tracts that had a higher percentage of the population living below the poverty level (AOR 0.51, 95% CI 0.28-0.92).

**Conclusions:**

We used spatial epidemiology and statistical modeling to identify and characterize HIV hotspots for the general population, MSM, and African Americans. HIV clusters concentrated in Jackson and the Mississippi Delta. African American race and urban location were positively associated with clusters, whereas having less than a high school education and having a higher percentage of the population living below the poverty level were negatively associated with clusters. Spatial epidemiological analyses can inform implementation science and public health response strategies, including improved HIV testing, targeted prevention and risk reduction education, and tailored preexposure prophylaxis to address HIV disparities in the South.

## Introduction

In the United States, HIV infections cluster geographically [1,2]. New HIV infections are concentrated in the Southeast and in neighborhoods with high rates of poverty that are predominately Hispanic/Latino or African American [2,3]. Geographic information system (GIS) and spatial epidemiological analyses provide opportunities to better understand the spatial distribution of HIV/AIDS, high-risk areas for disease transmission and acquisition [4], access to prevention interventions [5], and “hotspot” clustering of HIV-related mortality [6]. Understanding geographic clustering of HIV infections can also be helpful for directing limited public health resources to communities that bear a disproportionate share of HIV disease burden. Spatial epidemiological and geostatistical analyses can facilitate identification and characterization of HIV hotspot clusters down to the neighborhood level [6], which can be useful for targeting HIV prevention and care interventions in communities with high rates of HIV infection, and specific sociodemographic characteristics associated with clustering.

The southern United States accounted for 52% of HIV infections in 2014 [7]. Mississippi ranks ninth highest in rate of new HIV infections [2] and had the eighth-highest AIDS death rate of any state in the United States in 2016 [8]. Mississippi has alarming sociodemographic and geographical disparities related to HIV/AIDS; African Americans comprise only approximately 38% of the total population, but accounted for 80% of diagnosed HIV cases in 2014 [9]. Additionally, Jackson, Mississippi, had the fifth-highest AIDS diagnosis rate in 2014 and ranked fourth for HIV infection among metropolitan areas in the United States. Moreover, from 2005 to 2014, the number of infections among men who have sex with men (MSM) in Mississippi increased 59%, with an even sharper increase among young African American MSM [9,10]. A recent study found that Jackson has the highest rate of new HIV infections among MSM of any city nationwide [11]. Further, a recently completed study of 609 young African American MSM residing in Jackson observed that 27.9% were HIV infected at baseline and 6.8% of the remainder acquired HIV during the ensuing 12 months [12].

The goal of this study was to identify and characterize HIV hotspot clusters in Mississippi. We aimed to test the hypotheses that HIV infections clustered geospatially in Mississippi and that HIV hotspots were associated with sociodemographic factors (eg, race, income, education) in local communities.

## Methods

### Data Sources

We obtained deidentified HIV surveillance data from the Mississippi State Department of Health. The dataset included newly reported HIV cases from 2008 to 2014 (N=3410) with variables such as race, ethnicity, age at diagnosis, year of diagnosis, sex at birth, sexual orientation, and the census tract of residence at the time of diagnosis. The HIV cases were geocoded by the Mississippi State Department of Health. Among the 3410 reported cases, 2732 (80.12%) had a census tract of residence identified (ie, geocoded). In comparative analyses, we did not find any significant differences between reported cases that had a census tract of residence versus those that did not. Cases identified in prisons and decedent cases were excluded from the dataset, resulting in a total of 2048 cases. These 2048 georeferenced cases were aggregated at the census tract level to facilitate spatial analyses and creation of GIS maps, while protecting the confidentiality of people living with HIV.

We also obtained population denominators and community-level sociodemographic data, on the census tract level, from the US Census Bureau’s American Community Survey (ACS) 2010-2014 Five-Year Estimates [13]. In the 2010 Census, there were 664 census tracts in Mississippi. Using the surveillance and ACS data, we calculated HIV rates per 100,000 people at the census tract level. Census tracts with a total population of less than one were deemed as “uninhabited” and were excluded from all analyses, resulting in an omission of six tracts in Mississippi. Our final analytical dataset included HIV rates and community-level covariates for 658 Mississippi census tracts.

### Geographic Information System Mapping and Spatial Analysis

First, we created descriptive GIS maps to determine the initial spatial distribution of HIV cases and rates across all Mississippi census tracts. We categorized the HIV counts and rates by quintiles in thematic maps to create cut points at the 20th, 40th, 60th, and 80th percentiles. The lowest quintile represented HIV levels from zero to the 20th percentile (lowest HIV burden) and the highest quintile represented HIV levels for census tracts that fell between the 80th to 100th percentile (highest HIV burden). Initial descriptive maps for distinct study years did not depict substantial variation across years. Aggregated data across all study years also provided greater statistical power to identify clusters. Because we had access to data for our outcomes and covariates at the census tract level (rather than the address level), we opted to use a five-step spatial analytical approach to identify HIV hotspots:

1. First, we temporarily excluded large census tracts (outliers that were >1.5 standard deviations above the mean square mile area for all census tracts in Mississippi) that might bias distance calculations in subsequent steps.
2. Next, we calculated the spatial connectivity of the census tracts in Mississippi by calculating the mean and maximum distance between the geocentroid (ie, the geographic center) of each census tract and the geocentroids of the two nearest neighboring census tracts to obtain distance parameters for the next step.
3. We conducted incremental spatial autocorrelation analyses to determine the distance at which clustering for our outcome of interest was most intense (ie, we identified the most significant spatial sphere of influence with regard to HIV clustering in Mississippi) [14].
4. We calculated a spatial weights matrix that accounted for the spatial relationships of all census tracts, including the large census tracts excluded from previous steps, and our HIV measures (ie, counts and rates), improving the validity of the hotspot analysis.
5. We calculated the Getis-Ord Gi* statistic, which produces *z* scores, to identify clustering patterns across all Mississippi census tracts. Ultimately, a census tract was identified as belonging to a hotspot (or coldspot) cluster when it, and its neighboring census tracts, had a local mean HIV count/rate that was higher or lower than the mean HIV rate for all census tracts in Mississippi (ie, when the local mean HIV rate was higher or lower than the global mean HIV rate).

We have described hotspot analyses in more detail elsewere [15,16]. All maps and spatial analyses were conducted in ArcGIS v10.3.1 (ESRI, Redlands, CA, USA).

### Statistical Analyses

We assessed measures of central tendency (ie, mean, median, 95% confidence interval, and interquartile ranges) for all community-level sociodemographic explanatory variables. We calculated these descriptive statistics for all census tracts in Mississippi (n=658), census tracts located within HIV hotspot clusters (n=160), and census tracts outside of HIV hotspots (ie, tracts with mean HIV rates and coldspot clusters; n=498). Based on ACS data from the US Census Bureau, we created dichotomous categorical variables for the percentage of the population that was male and female, median annual individual income, median annual household income, percentage white, percentage African American, percentage Hispanic/Latino ethnicity, percentage Asian, percentage Hawaiian/Pacific Islander, percentage other races/ethnicities, percentage with two or more ethnicities, percentage of households on food stamps, and population density. Based on the statistical distribution of the data, we created trichotomous categorical variables with cut points at the 33rd and 66th percentiles for the percentage of population living 100% below the poverty level, families with two or more workers, and families with one person working in the household.

We used bivariate logistic regressions to assess crude associations between sociodemographic factors and HIV hotspot clusters (yes/no) on the census tract level. Associations with a *P*<.25 were included in the multivariable logistic regression models. A variance inflation factor greater than 6 indicated collinearity and led to exclusion of variables from the multivariable logistic regressions. We tested interactions for each of the race variables and the education variables: high school graduate or higher, and less than a high school education. In our final adjusted model, we considered associations with a *P*<.05 as statistically significant. Statistical analyses were conducted in SAS 9.3 (Cary, NC, USA).

## Results

**Figure 1:**
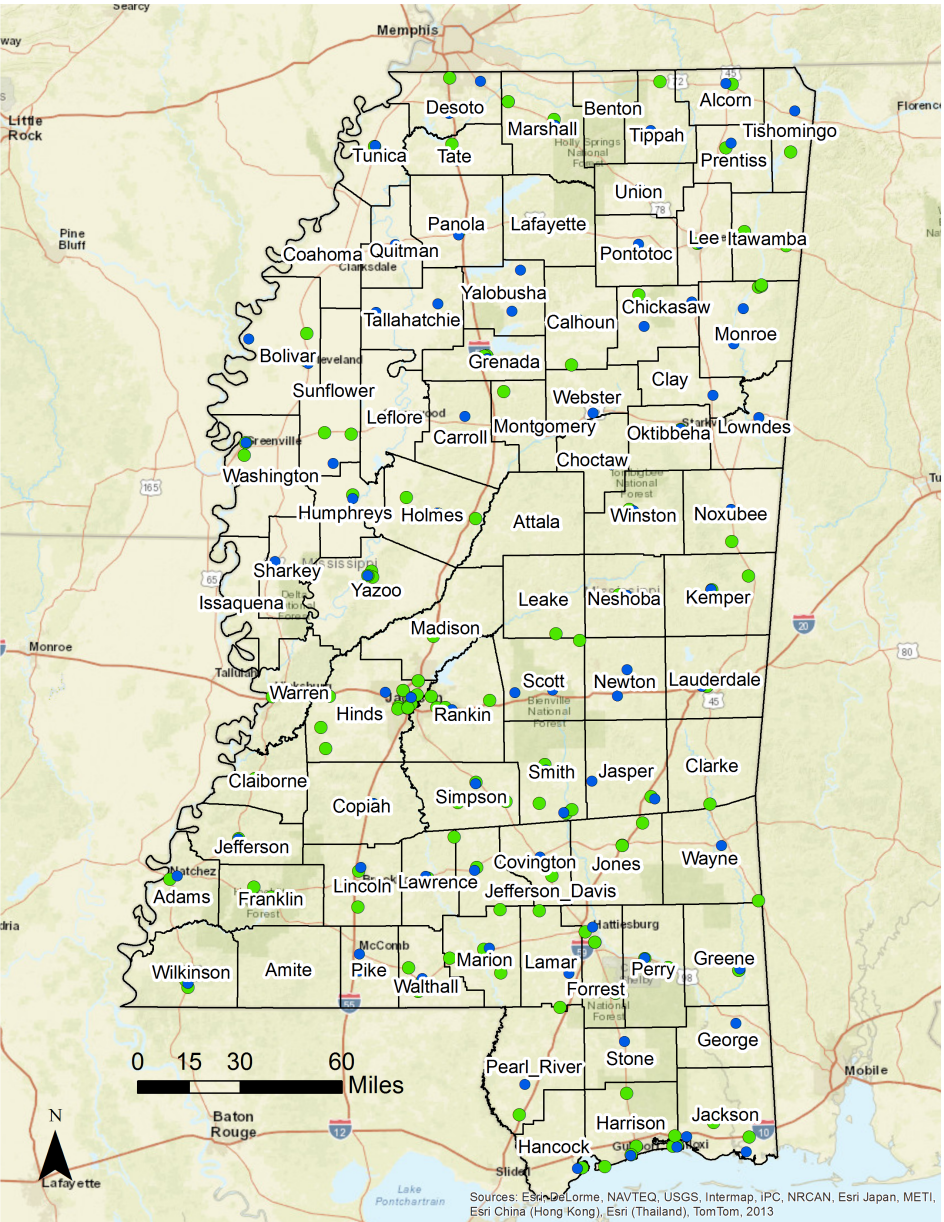
Locations of free HIV testing services that receive support for HIV screening from the Mississippi State Department of Health, 2014. Green points represent Ryan White clinics; Blue points represent County Health Departments.

Between 2008 and 2014, 3410 HIV cases were reported to the Mississippi Department of Health HIV surveillance system. Of these, 80.12% (2732/3410) had a census tract of residence and were successfully geocoded. In comparative analyses, we did not find any significant differences between reported HIV cases that had a census tract of residence versus those that did not. Typical reasons for lack of a geocoded case were missing address, incomplete address, and inclusion of a PO box as an address. Cases identified in prisons and decedent cases were excluded from the dataset, resulting in a total of 2048 cases in our final analytical dataset. These 2048 georeferenced cases were aggregated at the census tract level (n=658) to facilitate spatial analyses and creation of GIS maps. Figure 1 includes a reference map of Mississippi that portrays county boundaries, major highways, and locations with free HIV testing services, including Ryan White clinics and County Health Departments that receive support for HIV screening from the Mississippi State Department of Health.

**Figure 2:**
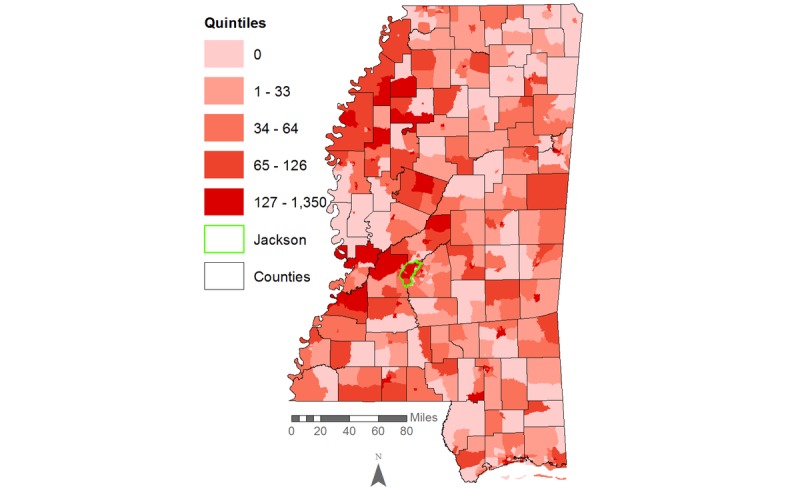
HIV rates per 100,000 population in Mississippi, 2008-2014.

**Figure 3:**
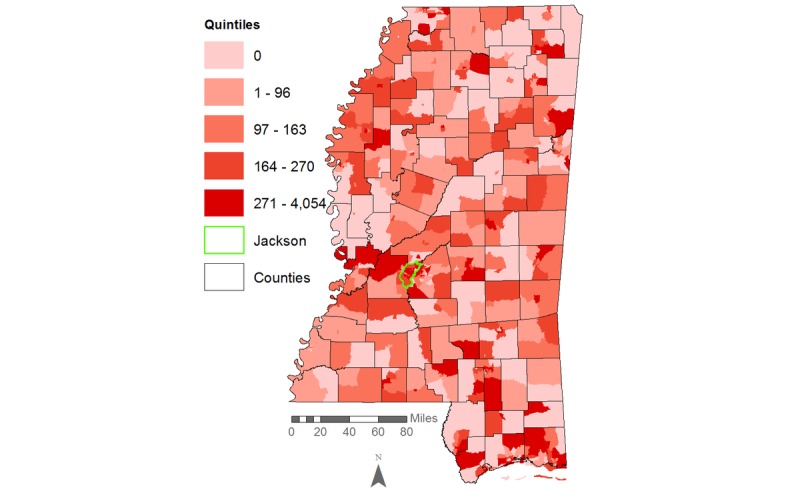
HIV rates per 100,000 African Americans in Mississippi, 2008-2014.

Our initial descriptive maps showed high HIV rates per 100,000 people dispersed throughout many regions of Mississippi, with rates of up to 127 to 1350 cases per 100,000 in the Jackson area for the entire population (Figure 2) and up to 271 to 4054 HIV cases per 100,000 among African Americans alone (Figure 3). Some census tracts in nonurban areas also had rates of HIV infection that fell between the 80th and 100th percentile (ie, the upper quintile).

**Figure 4:**
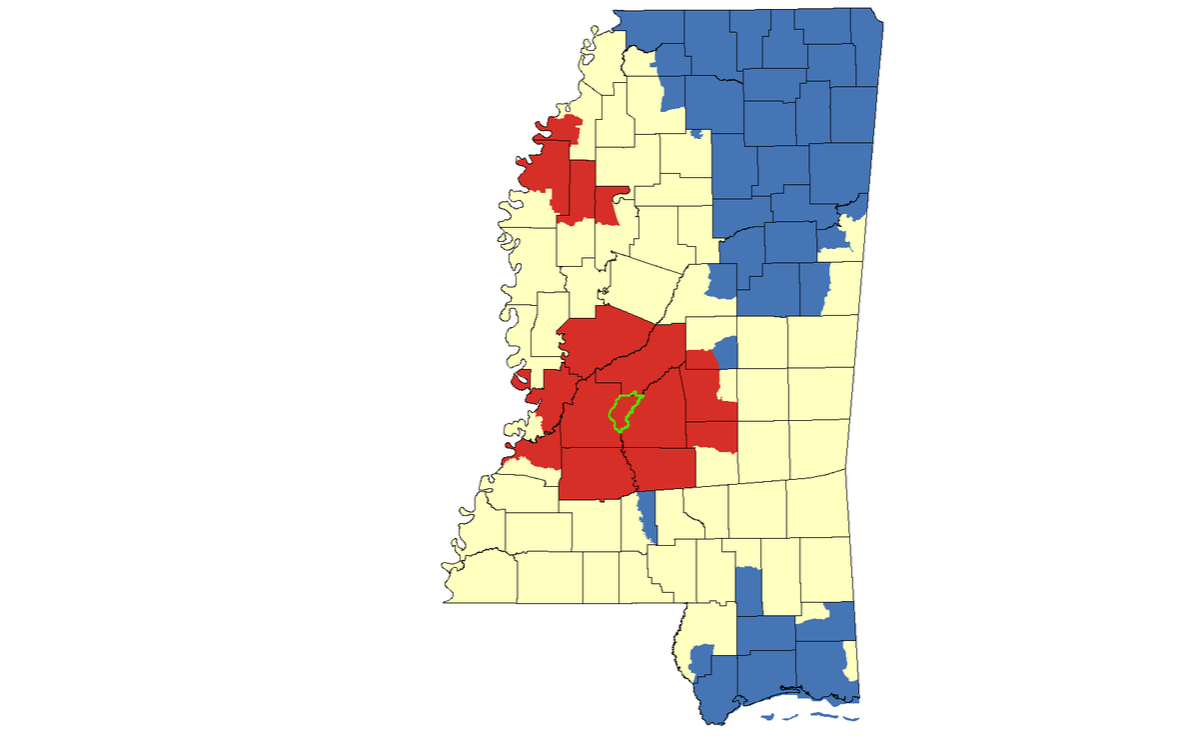
Hotspot cluster map for HIV rates per 100,000 population in Mississippi, 2008-2014. Clusters are based on HIV rates aggregated at the census tract level. Census tracts with elevated HIV rates (red) represent hotspots (P<.05); census tracts with low HIV rates (blue) represent coldspots (P<.05); census tracts with mean HIV rates are represented in yellow. The Jackson Metropolitan Area is outlined in green.

**Figure 5:**
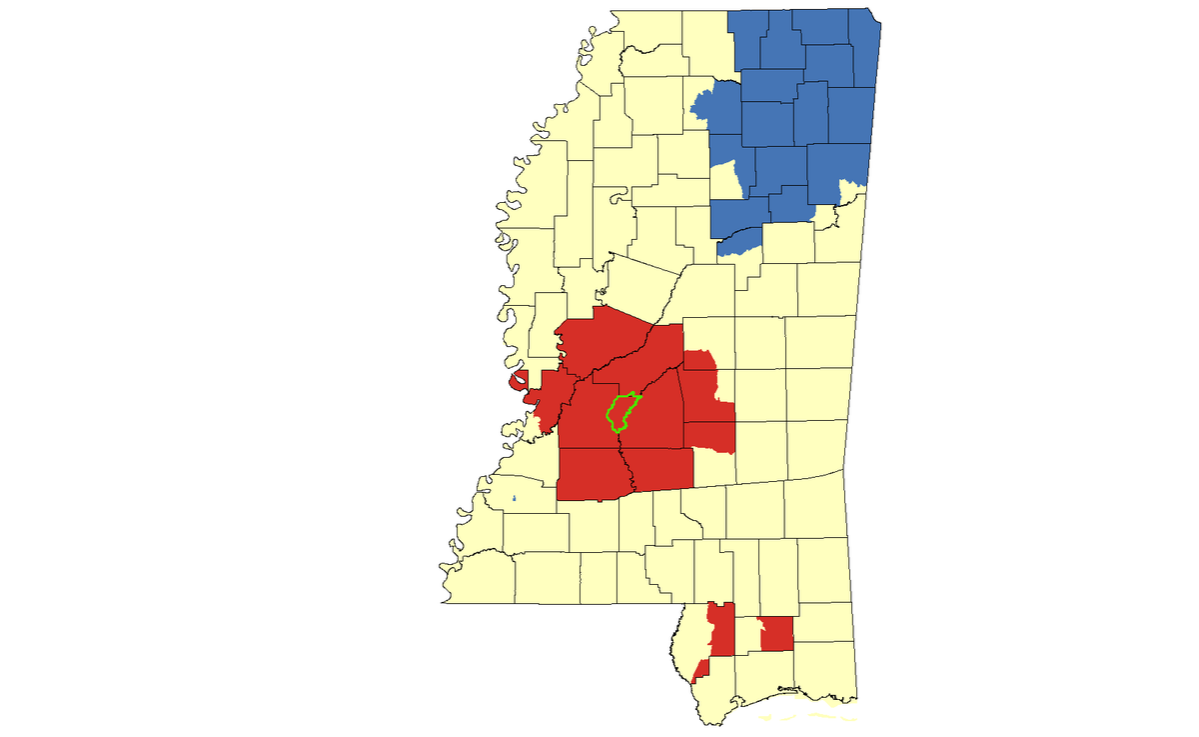
Hotspot cluster map for HIV rates per 100,000 African Americans in Mississippi, 2008-2014. Clusters are based on African American HIV rates aggregated at the census tract level. Census tracts with elevated African American HIV rates (red) represent hotspots (P<.05); census tracts with low African American HIV rates (blue) represent coldspots (P<.05); census tracts with mean African American HIV rates are represented in yellow. The Jackson Metropolitan Area is outlined in green.

Results from our hotspot cluster analyses indicated that there was a large statistically significant hotspot cluster for HIV rates in Jackson and surrounding counties, with smaller hotspots in the Mississippi Delta region, including Cleveland, Bolivar, Sunflower, Leflore, Coahoma, and Tallahatchie Counties (Figure 4). Census tracts in the greater Jackson area also appeared in HIV hotspot clusters when the analysis was limited to African Americans. Smaller HIV clusters in Pearl River and Stone Counties were also identified for African Americans (Figure 5).

**Figure 6:**
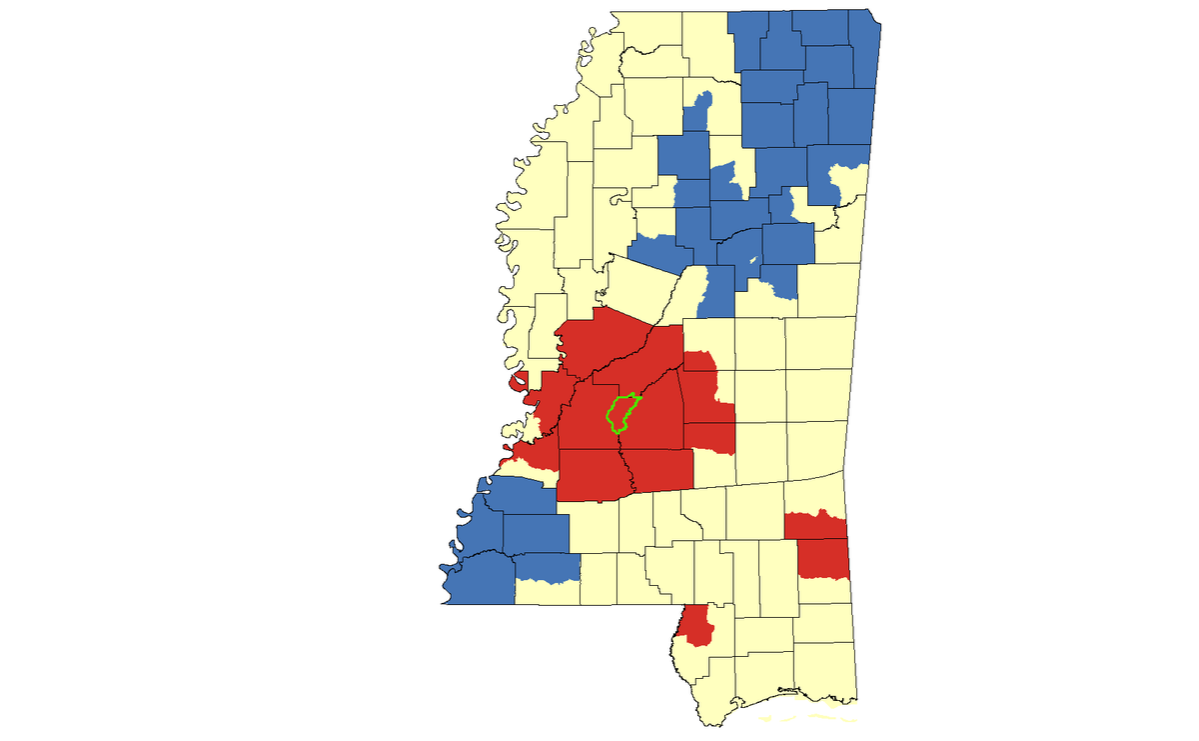
Hotspot cluster map for HIV case counts among MSM in Mississippi, 2008-2014. Clusters are based on MSM case counts aggregated at the census tract level. Census tracts with elevated numbers of MSM living with HIV (red) represent hotspots (P<.05); census tracts with low numbers of MSM living with HIV (blue) represent coldspots (P<.05); census tracts with mean numbers of MSM living with HIV are represented in yellow. The Jackson Metropolitan Area is outlined in green.

**Figure 7:**
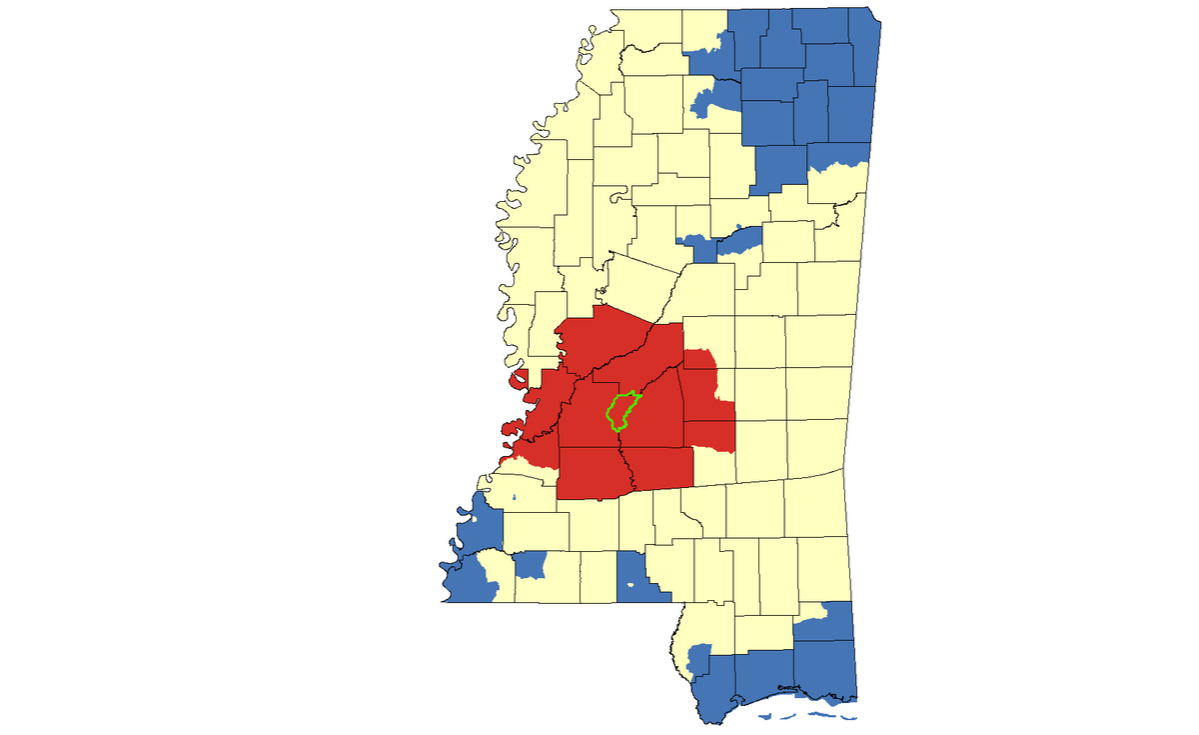
Hotspot cluster map for HIV case counts among African American MSM in Mississippi, 2008-2014. Clusters are based on African American MSM case counts aggregated at the census tract level. Census tracts with elevated numbers of African American MSM living with HIV (red) represent hotspots (P<.05); census tracts with low numbers of African American MSM living with HIV (blue) represent coldspots (P<.05); census tracts with mean numbers of African American MSM living with HIV counts are represented in yellow. The Jackson Metropolitan Area is outlined in green.

In our cluster analysis focused on HIV cases among MSM, we again identified the greater Jackson area as a hotspot, and we identified smaller hotspots in Pearl River County and Greene/Wayne counties (Figure 6). When we limited our cluster analysis to only those HIV cases identified as both African American and MSM, we again identified a hotspot in the greater Jackson area (Figure 7).

**Table 1:** Descriptive statistics of Mississippi census tracts, 2010-2014 (n=658).

Characteristic		Census tracts in HIV hotspots, mean (95% CI) (n=160)	Census tracts outside HIV hotspots, mean (95% CI) (n=498)
**Sex (%)**			
	Male	48.0 (47.1-48.8)	48.78 (48.3-49.2)
	Female	52.1 (51.2-52.9)	51.2 (50.8-51.7)
Total population		4410.7 (4052.4-4768.9)	4584.2 (4415.3-4753)
**Median age (years)**		36.1 (35.1-37.0)	37.31 (36.8-37.8)
	<5	6.9 (6.5-7.3)	6.7 (6.5-6.9)
	5-17	18.4 (17.6-19.1)	17.7 (17.4-18.1)
	18-24	10.3 (9.4-11.3)	10.6 (10.0-11.3)
	25-44	26.3 (25.4-27.4)	24.9 (24.5-25.3)
	45-54	13.5 (13-14)	13.5 (13.2-13.7)
	55-64	12.1 (11.5-12.7)	12.3 (12.0-12.6)
	65-74	7.1 (6.6-7.5)	8.2 (7.9-8.5)
	≥75	5.4 (5.1-5.8)	6.2 (6.0-6.4)
**Race/Ethnicity (%)**			
	White	43.7 (39-48.5)	59 (56.7-61.3)
	African American	53.5 (48.6-58.3)	37.6 (35.3-39.9)
	Hispanic	2.4 (1.8-3.1)	3.0 (2.6-3.3)
	Asian	0.98 (0.71-1.3)	0.81 (0.67-0.96)
	Hawaiian/Pacific Islander	0.01 (0-0.02)	0.02 (0.01-0.03)
	Other race^a^	0.94 (0.58-1.3)	0.76 (0.61-0.96)
	Two or more races/ethnicities	0.66 (0.54-0.78)	1.3 (1.1-1.5)
Less than very well-spoken English (%)		1.8 (1.3-2.3)	1.5 (1.3-1.7)
**Education (%)**			
	Less than high school education	16.9 (15.3-18.5)	20.0 (19.3-20.8)
	High school graduate or higher	83.1 (75.6-84.4)	78.0 (79.2-80.7)
Median individual income (US$)		23,780 (22,274-25,286)	20,498.8 (19,970-21,027.6)
**Socioeconomic measures (%)**			
	Below 100% of the poverty level	10.3 (8.9-11.6)	12.1 (11.3-12.9)
	Population per square mile	1224 (1020-1429)	619.3 (537.1-701.4)
	Own housing (%)	63.8 (60.6-67.1)	67.1 (65.5-68.7)
	Rent housing (%)	36.2 (32.9-39.4)	32.7 (31.1-34.3)
	Food stamps (%)	19.3 (17.1-21.5)	20 (19-21)
	Households below poverty level (%)	22.3 (20.2-24.5)	23.5 (22.5-24.5)
	Household median income (US$)	43,591 (40,090-47,092)	37,398.9 (36,210-38,588)
	Families with one worker (%)	37.7 (36.4-39.1)	37.9 (37.1-38.6)
	Families with two or more workers (%)	45.1 (43.1-47)	41.8 (40.8-42.7)
	Urban area	0.36 (0.28-0.43)	0.16 (0.13-0.19)

^a^ Other race includes Asian, Hawaiian, Pacific Islanders, and other races/ethnicities not defined as white, African American, or Hispanic.

**Table 2:** Factors associated with HIV hotspots, Mississippi, 2008-2014 (n=658).

Characteristic		Unadjusted model, OR (95% CI)	Adjusted model 1,^a^ AOR^b^ (95% CI)	Adjusted model 2,^c^ AOR (95% CI)
**Male (%)**				
	<48.25	Referent	Referent	Referent
	≥48.25	0.70 (0.49-1.00)	0.94 (0.64-1.40)	0.94 (0.63-1.40)
**Female (%)**				
	<51.70	Referent	Referent	Referent
	≥51.70	1.43 (0.99-2.05)	—^d^	—
**Total population**				
	<4199	Referent	Referent	Referent
	≥4199	0.79 (0.55-1.13)	0.85 (0.55-1.31)	0.85 (0.55-1.32)
**Median age (years)**		0.96 (0.93-0.99)	0.96 (0.92-1.00)	0.95 (0.91-1.00)
	<5	1.04 (0.96-1.12)	—	—
	5-17	1.04 (0.99-1.08)	—	—
	18-24	0.99 (0.97-1.02)	—	—
	25-44	1.06 (1.02-1.10)	—	—
	45-54	1.01 (0.95-1.07)	—	—
	55-64	0.98 (0.93-1.04)	—	—
	65-74	0.86 (0.80-0.92)	—	—
	≥75	0.89 (0.82-0.96)	—	—
Less than a high school education (%)		0.96 (0.94-0.98)	0.95 (0.92-0.98)	0.91 (0.86-0.96)
Low ability to speak English (%)		1.03 (0.97-1.10)	—	—
**White (%)**				
	<59.8	Referent	Referent	Referent
	≥59.8	0.55 (0.38-0.79)	—	—
**African American (%)**				
	<35.4	Referent	Referent	Referent
	≥35.4	2.01 (1.39-2.90)	3.85 (2.23-6.65)	1.15 (0.42-3.17)
**Hispanic (%)**				
	<1.4	Referent	Referent	Referent
	≥1.4	0.77 (0.54-1.1)	0.86 (0.56-1.31)	0.84 (0.55-1.29)
**Other ethnicity (%)** ^e^				
	<0.6%	Referent	Referent	Referent
	≥0.6%	1.11 (0.77-1.58)	—	—
**Two or more race/ethnicities (%)**				
	<0.7	Referent	Referent	Referent
	≥0.7	0.61 (0.43-0.88)	0.46 (0.30-0.70)	0.46 (0.30-0.72)
**Population density**				
	<273	Referent	Referent	Referent
	≥273	2.72 (1.87-3.97)	1.64 (0.96-2.80)	1.47 (0.86-2.51)
**Median annual household income (US$)**				
	<36,775	Referent	Referent	Referent
	≥36,775	1.26 (0.88-1.8)	1.12 (0.6-2.09)	0.80 (0.39-1.64)
**Median annual individual income (US$)**				
	<20,083	Referent	Referent	Referent
	≥20,083	1.53 (1.07-2.12)	—	—
	Rent housing (%)	1.01 (1.0-1.019)	0.99 (0.97-1.01)	0.99 (0.97-1.01)
	Own housing (%)	0.99 (0.98-1.00)	—	—
**Households living at 100% below poverty status (%)**				
	<17.3	Referent	Referent	Referent
	17.3-28.2	0.58 (0.37-0.90)	0.51 (0.28-0.92)	0.61 (0.33-1.13)
	≥28.3	0.80 (0.52-1.22)	0.43 (0.19-0.99)	0.48 (0.20-1.17)
Households living below poverty level (%)		0.76 (0.53-1.08)	—	—
Urban area		2.89 (1.93-4.32)	2.01 (1.20-3.37)	2.06 (1.23-3.46)
≥ **2 workers in the family (%)**				
	<37.9	Referent	Referent	Referent
	37.9-47.1	0.95 (0.59-1.52)		
	≥47.2	1.78 (1.16-2.73)	—	—
**1 worker in family (%)**				
	<34	Referent	Referent	Referent
	34-40.6	0.79 (0.51-1.23)		
	≥40.7	0.96 (0.62-1.47)	—	—
**Households on food stamps (%)**				
	<17.76	Referent	Referent	Referent
	≥17.76	0.88 (0.61-1.25)	—	—
Interaction between African American and less than a high school education		—	—	1.09 (1.02-1.16)

^a^ Adjusted for percentage of the population living 100% below the poverty line, percentage of the total population that was male, median annual individual income, median annual household income, total population, percentage of total population that was African American, percentage of total population that was Hispanic, population density, households with two or more workers, median age, percentage of the total population that had less than a high school education, the percentage of the population that rented housing, and areas categorized as urban.

^b^AOR: adjusted odds ratio.

^c^ Adjusted for the same as in adjusted model 1 and including the interaction variable between African American race and less than a high school graduate education.

^d^ Indicates the variable was not statistically significant at a *P*<.25 level in bivariate analyses and therefore was not included in multivariable models.

^e^ Other race includes Asian, Hawaiian, Pacific Islanders, and other races/ethnicities not defined as white, African American, or Hispanic.

The results of our descriptive statistical analyses are presented in Table 1. A total of 160 Mississippi census tracts were located within statistically significant HIV hotspot clusters, and 498 census tracts were located outside of HIV hotspot clusters.

In bivariate analyses focused on HIV rates for the entire Mississippi population, we noted significant associations between HIV hotspots and age, with positive associations between the percentage of the population that was 25 to 44 years of age (OR 1.06, 95% CI 1.02-1.10), census tracts with higher percentages of African American residents (OR 2.01, 95% CI 1.39-2.90), population density, median annual income, and residing in an urban area (Table 2). Significant protective associations were found between HIV hotspots and having less than a high school education (OR 0.96, 95% CI 0.94-0.98), being white (OR 0.55, 95% CI 0.38-0.79), identifying as two or more race/ethnicities (OR 0.61, 95% CI 0.43-0.88), and living below the poverty level (OR 0.58, 95% CI 0.37-0.90).

In the multivariable logistic regression models, we found that HIV hotspot clusters were significantly more likely to include census tracts that had a higher proportion of African Americans (adjusted odds ratio [AOR] 3.85, 95% CI 2.23-6.65) and urban census tracts (AOR 2.01, 95% CI 1.20-3.37), while controlling for all other factors. Hotspots were less likely to include census tracts with less than a high school education (AOR 0.96, 95% CI 0.94-0.98), people identifying as belonging to two or more racial/ethnic groups (AOR 0.46, 95% CI 0.30-0.70), and having 17.3% to 28.3% of the population living 100% below the poverty level (AOR 0.51, 95% CI 0.28-0.92).

In the second adjusted model, we included the interaction term for African American race and education, and found statistically significant associations with HIV hotspots and having less than a high school education (AOR 0.91, 95% CI 0.86-0.96), identifying as two or more races/ethnicities (AOR 0.46, 95% CI 0.30-0.72), and being located in an urban area (AOR 2.06, 95% CI 1.23-3.46). In this model, we noted a decrease in statistically significant associations between HIV hotspot clusters and other covariates. Thus, we included both multivariable models in Table 2 for comparison.

## Discussion

### Principal Findings

In this set of analyses conducted at the neighborhood (ie, census tract) level, we found that several geographic, demographic, and community-level factors were correlated with HIV hotspot clustering in Mississippi. The focus on clustering of all HIV infections, and infections among MSM, African Americans, and African American MSM allowed us to observe overlap and unique patterns in clustering across different populations. Both our multivariable models and geospatial hotspot cluster mapping suggests that in this largely rural state, urban residence is an important risk factor for HIV acquisition; most hotspot clusters—for African Americans, MSM, and African American MSM—were concentrated within or near the Jackson metropolitan area (Figures 5-7; outlined in green). This is consistent with the proposition that HIV spreads more rapidly in dense, highly populated sexual networks [17]. Indeed, research suggests that sexual networks may play a critical role in hastening HIV transmission, particularly among sexual and gender minorities in the United States and in Jackson, Mississippi [18-22].

In addition, we also found hotspot clusters in rural counties in the Mississippi Delta region, including Cahoma, Bolivar, and Sunflower Counties in the Mississippi Delta (see Figure 3). In contrast with the Jackson metropolitan area, hotspot clusters were not present in rural counties when analyses focused on MSM, suggesting that HIV transmission in these rural hotspots is attributed to heterosexual HIV transmission or different types of sexual networks. Another possibility is that there may be an underreporting of MSM contact in the Delta area, a rural area with high rates of HIV stigma. Lastly, we identified HIV hotspots among African Americans in Southern Mississippi, which may indicate higher transmission risks in the Gulf Coast Area, particularly among individuals who self-identify as heterosexual. We are uncertain whether there are strong network connections between the Gulf Coast Area and Jackson, or whether other mechanisms are in play in this region (eg, visitors from outside Mississippi who could bring elevated transmission risk). We believe that the sexual networks formed in urban and rural locations are associated with HIV transmission. Although sexual networks in urban locations may have increased densities compared to those in rural areas, we believe that urban-rural movement within sexual networks are associated with transmission patterns. A previous study in Mississippi documented this urban-rural pattern with phylogenetic analysis [23]. However, more research is needed given the lack of data on this rural phenomenon.

Taken together, these findings support the work of others that found that “place” is an important determinant of HIV acquisition risk [1,24-27] and that HIV is often concentrated in discrete geographic areas. Our analyses and results build on the descriptive maps presented in publicly available HIV data systems such as AIDSVu [28], which present HIV counts and rates at the county level. Our hotspot cluster analyses at the census tract level highlight statistically significant clusters of HIV at a more granular level (ie, census tract), which can be useful for targeting HIV prevention and care interventions in communities with high rates of HIV infection. Moreover, these findings underscore the need for different types of interventions in different geographic areas and among different subpopulations. As opposed to a static statewide intervention response, this cluster analysis allows for the strategic redirection of intervention efforts based on need rather than an assumption that all areas of the state warrant equal resources for HIV prevention. The cluster analysis methodology we employed could also be useful for other southern states.

Community-level factors were also associated with HIV hotspots. First, census tracts with between 17% and 28% of the population living below the federal poverty level had lower odds of being in HIV hotspot clusters, even after controlling for race, education, and other sociodemographic variables. This finding is congruent with other studies exploring concurrent sexual partnerships in Jackson, Mississippi, that found that higher income and higher education were associated with higher rates of overlapping sexual partnerships [29]. Similar trends have been found in Sub-Saharan Africa [30,31]. However, a large body of research in the United States found that HIV prevalence is higher among low-income individuals and communities, highlighting the differential role that income may play in distinct social contexts [32,33]. Although the mechanisms through which income and education may affect HIV acquisition risks have been studied previously [34], they are not yet well understood in the Deep South. Our findings suggest that higher income and education may be associated with increased number of sexual partnerships as well as geographic and social mobility, both of which can influence inner-connectivity of sexual networks. Low levels of income may also limit access to technology such as internet, mobile phones, and hookup apps, which can also expedite meeting of sexual partners and mobility within networks.

Most HIV prevention and care services in Mississippi are concentrated in Jackson. Further, most HIV screening that took place in 2015 in Mississippi was sponsored by the Mississippi Department of Health. Other parts of Mississippi have limited prevention and care services; far fewer services are delivered outside of Jackson. Larger sexual networks and limited access to HIV screening, treatment, and prevention services in urban geographic hotspots of infection likely compound HIV risks, particularly among MSM. Our findings underscore the need for expanding and tailoring HIV prevention and care services to the populations in urban hotspot clusters as well as less urbanized areas of the Mississippi Delta and the Gulf Coast Region. Expanding the density of HIV testing locations to correspond with the identified hotspots is one important policy implication of the study findings.

These findings have important implications for implementation science and the public health response agenda focused on reducing racial and geographic disparities in HIV infection in the Deep South. Implementation science studies should explore how best to scale up existing HIV prevention, treatment, and care interventions in geographic hotspots in the Jackson metropolitan area, as well as in rural communities. Our results also suggest that culturally congruent interventions are likely important for different geographic regions: interventions in the Jackson metropolitan area should have a keen focus on engaging African American MSM in HIV prevention and care services, whereas interventions in the Delta may need greater focus on reaching heterosexual populations or reaching MSM who may not self-identify as gay.

A study of primary care physicians in the Mississippi Delta region suggests that expanding HIV screening to improve access is feasible [35]. Taken together, these findings underscore the urgency of engaging primary care providers in the Mississippi Delta effort to prevent HIV. Mississippi also has a robust effort to expand telemedicine, which could enhance access to HIV care for individuals in rural areas [36].

### Limitations

Our study is subject to several limitations. First, a growing body of research suggests that complex sexual networks, particularly among African American MSM, contribute to HIV acquisition risks in Mississippi. However, surveillance data on HIV risk behaviors and sexual networks are not available and were therefore not included in our models. The surveillance data we used consisted of newly reported cases rather than incident cases; therefore, the HIV hotspots we identified do not necessarily reflect hotspots of recent HIV infection. Additionally, the location of HIV testing services may influence the location where cases are reported: fewer cases may be reported from an area located farther away from testing services because fewer people can access testing. We were able to geocode 80% of HIV cases in the Mississippi HIV surveillance data. Although this geocoding rate decreased the number of HIV cases that were included in our analyses, it is comparable to geocoding rates achieved in other studies focused on small area analysis in geospatial research [37]. Further, comparison of HIV cases that geocoded versus those that did not geocode were not significantly different providing further evidence that the data we analyzed are representative of the HIV epidemic in Mississippi between 2008 and 2014. Our results are based on ecological analyses. We did not have access to address-level data for the place of residence for HIV cases. Nonetheless, we were able to provide descriptive maps and cluster analysis results down to the census tract level, which can help to provide public health officials with a more detailed understanding of neighborhood-level geographic distribution of HIV clusters. Individual-level measures from the surveillance data were used to conduct spatial analyses focused on specific subgroups by race, sex, and age. Future research may benefit from multilevel hierarchical statistical models.

### Future Directions

These study findings add to a mounting body of evidence underscoring how geographic location, community-level factors, and sexual orientation may impact HIV acquisition risks in the Deep South of the United States. We now have a list of important HIV prevention tools to deploy to reduce HIV transmission, including HIV testing, treatment as prevention, preexposure prophylaxis, and condom use. Our results could, for instance, help guide the locations where a mobile HIV testing clinic or a mobile clinic to prescribe preexposure prophylaxis could be positioned to address the local needs within hotspots. Our findings suggest a greater need for public health programs and implementation science research that explore how to effectively deploy these tools in “hotspot” communities in the Deep Southern United States with high rates of HIV infection and limited prevention, treatment, and care services.

## Abbreviations

ACS: American Community Survey
AOR: adjusted odds ratio
GIS: geographic information system
MSM: men who have sex with men
